# Salmonella Phages Affect the Intestinal Barrier in Chicks by Altering the Composition of Early Intestinal Flora: Association With Time of Phage Use

**DOI:** 10.3389/fmicb.2022.947640

**Published:** 2022-07-14

**Authors:** Hongze Zhao, Yue Li, Peilin Lv, Jinmei Huang, Rong Tai, Xiue Jin, Jianhua Wang, Xiliang Wang

**Affiliations:** ^1^State Key Laboratory of Agricultural Microbiology, College of Veterinary Medicine, Huazhong Agricultural University, Wuhan, China; ^2^College of Veterinary Medicine, Huazhong Agricultural University, Wuhan, China; ^3^Hubei Provincial Institute of Veterinary Drug Control, Wuhan, China

**Keywords:** chicks, phage, intestinal barrier, cecal microbiota, *Enterococcus*

## Abstract

Phages show promise in replacing antibiotics to treat or prevent bacterial diseases in the chicken breeding industry. Chicks are easily affected by their environment during early growth. Thus, this study investigated whether oral phages could affect the intestinal barrier function of chicks with a focus on the cecal microbiome. In a two-week trial, forty one-day-old hens were randomly divided into four groups: (1) NC, negative control; (2) Phage 1, 10^9^ PFU phage/day (days 3–5); (3) Phage 2, 10^9^ PFU phage/day (days 8–10); and (4) AMX, 1 mg/mL amoxicillin/day (days 8–10). High-throughput sequencing results of cecal contents showed that oral administration of phages significantly affected microbial community structure and community composition, and increased the relative abundance of *Enterococcus*. The number of different species in the Phage 1 group was much higher than that in the Phage 2 group, and differences in alpha and beta diversity also indicated that the magnitude of changes in the composition of the cecal microbiota correlated with the time of phage use. Particularly in the first stage of cecal microbiota development, oral administration of bacteriophages targeting *Salmonella* may cause substantial changes in chicks, as evidenced by the results of the PICRUSt2 software function prediction, reminding us to be cautious about the time of phage use in chicks and to avoid high oral doses of phages during the first stage. Additionally, the Phage 2 samples not only showed a significant increase in the relative abundance of *Bifidobacterium* and *Subdoligranulum*, but also improved the intestinal morphology (jejunum) and increased the mRNA expression level of occludin and ZO-1. We concluded that phages do not directly interact with eukaryotic cells. The enhancement of intestinal barrier function by phages in chicks may be related to changes in the intestinal flora induced by phages. This implies that phages may affect intestinal health by regulating the intestinal flora. This study provides new ideas for phage prevention of intestinal bacterial infections and promotes large-scale application of phages in the poultry industry.

## Introduction

Studies on intestinal microbiota have provided rigorous experimental evidence that the gut microbiota directly and indirectly modulates gut barrier function, digestion, host metabolism, and immune functions (Ivanov et al., 2009; Vijay-Kumar et al., 2010; Bischoff et al., 2014). Large-scale chicken farms are susceptible to bacterial diseases, especially during the early stages of growth. One of the reasons chicks are more susceptible to bacterial diseases than adult chickens is that incompletely developed intestinal microbes do not provide adequate protection against pathogen invasion (Hu et al., 2018). The improvement of the intestinal flora is one of the reasons why antibiotics are used as feed additives and are an important means of treating bacterial diseases (Banerjee et al., 2018). However, bacterial resistance and dysbiosis caused by the misuse of antibiotics, which also affects the economic efficiency of chickens, have become common concerns (Francino, 2015). Therefore, a new antibiotic alternative is needed to strengthen the prevention and treatment of bacterial diseases in chicks.

Lytic phages are viruses that selectively infect and kill bacteria. Phages were discovered in the early 20th century and were subsequently used to treat bacterial infections in human patients. Although long overshadowed by antibiotics, phages have garnered renewed interest worldwide because of the rising frequency of multidrug-resistant bacteria (Chanishvili, 2012; Golkar et al., 2014). Compared to antibiotics, phages have multiple advantages, including replicability, rapid sterilization, low inherent toxicity, low risk of resistance induction, and especially, high specificity (Abedon et al., 2011; Chan et al., 2013; Reardon, 2014; Gordillo Altamirano and Barr, 2019; Kortright et al., 2019), indicating minimal disruption to the normal flora (Gindin et al., 2019). Many researchers have demonstrated that the ability of phages to precisely regulate the microbiome can help in the treatment of diseases. Studies have shown that in chicks, bacteriophage predation of *Campylobacter jejuni* selectively reduces the relative abundance of *C. jejuni* without affecting the microbiota structure (Richards P. J. et al., 2019). A commercial cocktail of *Escherichia coli* bacteriophages selectively reduced *E. coli*, leading to an increase in abundance of the butyrate-producing genus (*Eubacterium*) and a decrease in the proportion of taxa most closely related to *Clostridium perfringens* (Febvre et al., 2019). Additionally, *Salmonella* bacteriophage CKT1 can improve the growth performance of chicks by alleviating disturbance to the intestinal flora by *Salmonella Pullorum* (Huang et al., 2022). These results suggest that phages can regulate intestinal health without destroying the intestinal flora.

The concept of improving animal health through improved gut health has existed in animal food production for decades. Thus, the development of gut microbiota plays a major role in poultry health, productivity, and disease control (Montoro-Dasi et al., 2020). During the period of gut microbial development, chicks are susceptible to infection with bacteria such as *Salmonella*; therefore, we plan to apply *Salmonella*-targeting phages for prevention. However, there is a paucity of information on the effects of treatments targeting the gut environment on the intestinal microbiome of chicks (Oakley et al., 2014; Waite and Taylor, 2014). Considering the critical role of phages in precise regulation of the microbiome, we hypothesized that *Salmonella*-targeting phages do not destroy the cecal flora of chicks and may positively regulate the intestinal flora. Here, we addressed this hypothesis in an experimental chicken model by sequencing of the V3–V4 region of the 16S ribosomal RNA (rRNA) gene to compare the microbiota composition and detect changes in intestinal barrier function before and after bacteriophage challenge at different stages of cecal microbiota development. This study aimed to determine the internal relationship between oral phages and intestinal barrier function and provides a basis for the application of phages in chicken breeding programs.

## Materials and Methods

### Phages Propagation and Purification

The bacteriophage GRNsp6 (GenBank accession ON526838) was isolated from chicken manure samples from a chicken farm (Jiangsu, China) and stored in the Laboratory of Veterinary Microbiology and Immunology, Huazhong Agricultural University.

The amplification of bacteriophages in liquid culture was based on Skaradzińska’s method (Skaradzińska et al., 2020) and the host strain of the bacteriophage GRNsp6 was ATCC13076. The phages were concentrated and purified using PEG8000 and cesium chloride (CsCl) density gradient ultracentrifugation according to the methods (Bourdin et al., 2014; Luong et al., 2020). The phage particles were re-suspended using sterile phosphate-buffered saline (PBS; pH = 6.2). Additionally, a gradient was produced using 1.30 g/cc, 1.50 g/cc, and 1.60 g/cc CsCl in a 14 × 89-mm ultracentrifuge tube. Lastly, we removed endotoxins through a Pierce High-Capacity Endotoxin Removal Spin 1 mL Column (Thermo Scientific, Japan) and then used a lyophilized amebocyte lysate assay (Xiamen Bioendo, Xiamen, China) for endotoxin detection. To accurately calculate the concentrations of phages using a single plaque assay, the agarose overlay technique was used (Abedon and Yin, 2009). The titer of the purified phage was 5 × 10^10^ PFU/mL, it did not change significantly when stored in PBS (pH = 6.2) at 4°C for 2 weeks, and the endotoxin level was approximately 50 EU/mL.

### Animal Experiment and Sample Collection

This study was approved by the Animal Experimental Ethical Inspection of the Laboratory Animal Center, Huazhong Agricultural University (HZAUCH-2022-0004). Forty healthy male Roman chicks (1-day-old, 30 ± 2 g) were purchased from salmonella-negative breeding farms and randomly divided into four groups, with ten chicks in each group. The negative control (NC) group was given 500 μL PBS (pH = 6.2)/day (days 3–5 and 8-10) by continuous gavage; the Phage 1 group was orally administered 500 μL 10^9^ PFU phage/day (days 3–5) and 500 μL PBS/day (days 8–10) by gavage; the Phage 2 group was administered 500 μL PBS/day (days 3–5) and 500 μL 10^9^ PFU phage/day (days 8–10) by gavage; and the AMX group was administered 500 μL PBS/day (days 3–5) and 500 μL 1 mg/mL amoxicillin (days 8–10) in the same way. The solvent for phages and amoxicillin was PBS (pH = 6.2) and the prepared 1 mg/ml amoxicillin solution was used immediately on preparation. Roman chicks were raised in a temperature-controlled and humidity-controlled room with four compartments of approximately 8 m^2^. Each group was housed in stainless steel cages with different compartments, the cages were 130 cm in width, 60 cm in length, and 70 cm in height. Chicks were raised under standard hygienic conditions (temperature, 35 ± 2°C; humidity, 65% ± 5). All animals had free access to food and water. The body weight of the chicks was recorded on days 3 and 14 of the trial. On the 14th day, five hens in each group were randomly selected for blood collection and the removal of duodenum, jejunum, and cecum contents. Serum was separated and used to analyze the serum concentrations of endotoxin. The intestinal tissues of the duodenum and jejunum were collected for hematoxylin and eosin (H&E) staining and real-time quantitative PCR (qPCR). Cecum contents were flash-frozen in liquid nitrogen for subsequent analysis. In addition, anal swabs were taken from the chicks after the experiment and no salmonella bacteria were isolated based on a previously described method (Mohammed and Dubie, 2022).

### Observation of Intestinal Morphology

After a series of dehydration, transparency, wax penetration, and embedding steps, the duodenum and jejunum tissues were sectioned into 5 μm slices, stained with H&E, and examined using an Olympus BX53 microscope (Olympus, Tokyo, Japan). Morphometry of intestinal villi and crypts was measured using the OlyVlA software.

### Determination of Endotoxin Concentration in Serum

Commercial ELISA kits (Jiancheng Bioengineering Institute, Nanjing, China) were used to quantify endotoxin levels in serum samples, according to the manufacturer’s instructions. The absorbance of the samples was read spectrophotometrically at 450 nm. Endotoxin levels were calculated using a standard endotoxin concentration curve.

### RNA Isolation and RT-qPCR

Total RNA from intestinal tissues was extracted using AG RNAex Pro reagent (Accurate Biology, China). Reverse transcription of 1,000 ng of RNA was conducted using the Evo M-MLV mix kit using gDNA Clean for qPCR (Accurate Biology, China). Real-time PCR amplification was performed using a Bio-Rad CFX384 real-time PCR System (Bio-Rad, United States) with SYBR Green Premix Pro Taq HS qPCR kit (Accurate Biology, China). The relative mRNA abundance of the selected genes was normalized to GADPH using the 2^–ΔΔCt^ formula. The primers used for qPCR are presented in Table 1.

**TABLE 1:** Sequences for real-time PCR primers.

Primer names	Forward	Reserve
*GAPDH*	GCACGCCATCACTATCTTCCA	CTCCACAACATACTCAGCACCT
*Occludin*	ACGGCAGCACCTACCTCAA	GGGCGAAGAAGCAGATGAG
*claudin- 1*	CATACTCCTGGGTCTGGTTGGT	GACAGCCATCCGCATCTTCT
*ZO-1*	GGTCAGCCAGATGTGGATTT	CCGAAGCATTCCATCTTCAT
*IL-1*β	ACTGGGCATCAAGGGCTACA	GCTGTCCAGGCGGTAGAAGA
*TNF-*α	GCCCTTCCTGTAACCAGATG	ACACGACAGCCAAGTCAACG
*IFN-*γ	CAACCTTCCTGATGGC GTGAA	CGCTGGATTCTCAAGTCGTTCAT

### 16S rRNA Gene Sequencing and Bioinformatics Analysis

Microbial community genomic DNA (gDNA) was extracted from the cecal content samples using the E.Z.N.A. ^®^ stool DNA kit (Omega Bio-tek, Norcross, GA, United States). The concentration of gDNA was quantified using NanoDrop 2000 (Thermo Fisher Scientific), and agarose gel electrophoresis was used to detect the quality of gDNA. The V3-V4 regions of the bacterial 16S rDNA sequences were amplified using primer pairs 338F (5′-ACTCCTACGGGAGGCAGCAG-3′) and 806R (5′-GGACTACHVGGGTWTCTAAT-3′). Purified PCR products were sequenced on the Illumina HiSeq2500 PE250 platform. Raw reads from the original DNA fragments were demultiplexed, quality-filtered using fastp version 0.20.0 (Chen et al., 2018), and merged using FLASH version 1.2.7 (Magoc and Salzberg, 2011). All quality-filtered reads were clustered into operational taxonomic units (OTUs) based on a 97% identity threshold by UPARSE (Stackebrandt and Goebel, 1994; Edgar, 2013). The taxonomy of each OTU representative sequence was analyzed using the RDP Classifier version 2.2 (Wang et al., 2007) against the 16S rRNA database (e.g., Silva v138) using a confidence threshold of 0.7. Non-metric multidimensional scaling (NMDS) was used to estimate beta diversity. Differences in the relative abundance of bacteria among the four groups were determined using the Kruskal–Wallis H test. Microbial function was predicted using PICRUSt 2. The predicted genes and their respective functions were aligned to the Kyoto Encyclopedia of genes and genomes (KEGG) database.

### Statistical Analysis

The results are expressed as the mean and SEM. One-way ANOVA was used to identify significant differences among the different groups.*p < 0.05, ^**^p < 0.01, ^***^p < 0.001, and ^****^p < 0.0001. The Kruskal–Wallis H test was used to analyze the differential flora and KEGG pathway between groups, with *p < 0.05; ^**^p < 0.01; and *p* < 0.1 as a trend. Data were combined from at least five independent experiments, unless stated otherwise.

## Results

### Effect of Phage on Body Weight

Throughout the experiment, all chicks survived without clinical changes in the intestinal tract, liver, spleen, kidney, or stool. The animals did not show an unkempt coat, poor mental status, or decreased activity. As shown in Table 2, no significant differences were detected in body weight on days 14 and average daily gain after bacteriophage or amoxicillin administration.

**TABLE 2:** Effect of phage on body weight of chicks.

	Treatments			
items	NC	Phage 1	Phage 2	AMX
body weight at day 3 (g)	35.18 ± 0.36	35.38 ± 0.27	34.52 ± 0.33	34.96 ± 0.29
body weight at day 14 (g)	104.88 ± 0.92	104.71 ± 1.20	106.53 ± 0.94	107.28 ± 1.22
	Average Daily Gain (g)			
3-14 days	6.34 ± 0.06	6.30 ± 0.12	6.55 ± 0.09	6.57 ± 0.09

*Values present means ± SEM (n = 10). NC, negative control; Phage 1, 10^9^PFU GRNsp6 (day 3-5); Phage 2, 10^9^PFU GRNsp6 (day 8-10); AMX, 1 mg/ml amoxicillin (day 8-10).*

### Intestinal Histology

**FIGURE 1:**
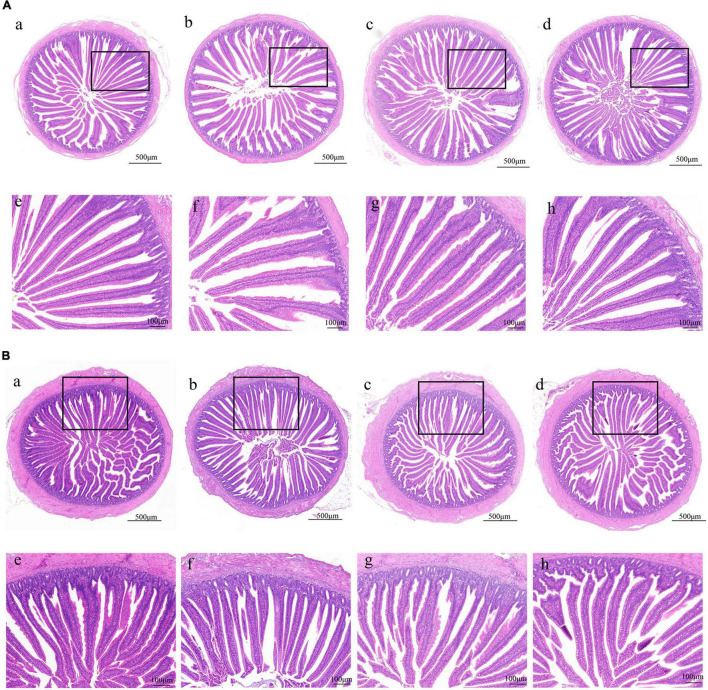
Effect of phages on the morphology of the duodenum and jejunum in chicks. **(A)** H&E staining of duodenum in the NC, Phage 1, Phage 2, and AMX groups under 40 × (a-d) and 100 × (e-h) magnification. **(B)** H&E staining of jejunum under 40 × (a-d) and 100 × (e-h) magnification. NC, negative control; Phage 1, 10^9^PFU GRNsp6 (day 3-5); Phage 2, 10^9^PFU GRNsp6 (day 8-10); AMX, 1 mg/ml amoxicillin (day 8-10).

**FIGURE 2:**
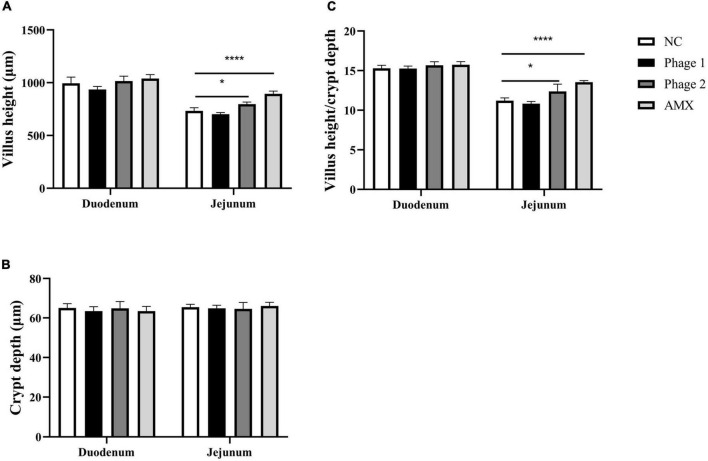
Effect of phages on **(A)** villus height, **(B)** crypt depth, and **(C)** villus height to crypt depth ratio of the duodenum and jejunum of chicks in the NC, Phage 1, Phage 2, and AMX groups, respectively. Values are means (n = 5). NC, negative control; Phage 1, 10^9^PFU GRNsp6 (day 3-5); Phage 2, 10^9^PFU GRNsp6 (day 8-10); AMX, 1 mg/ml amoxicillin (day 8-10). *p < 0.05; ****p < 0.0001.

Morphology, villus height, and crypt depth were assessed using H&E staining. The absorption capacity is positively related to the height of the intestinal villus and the ratio of villus height/crypt depth (V/C). Crypt depth responds to intestinal stem cell activity and function. As shown in Figure 1, the intestinal mucosa in all groups exhibited normal histology. Meanwhile, administration of bacteriophage (days 8–10) or amoxicillin significantly increased (*p* < 0.05) the height of villi and the V/C ratio in the jejunum, and we did not find a significant difference in crypt depth (Figure 2).

### Relative mRNA Expression of Jejunal Tight Junction

**FIGURE 3:**
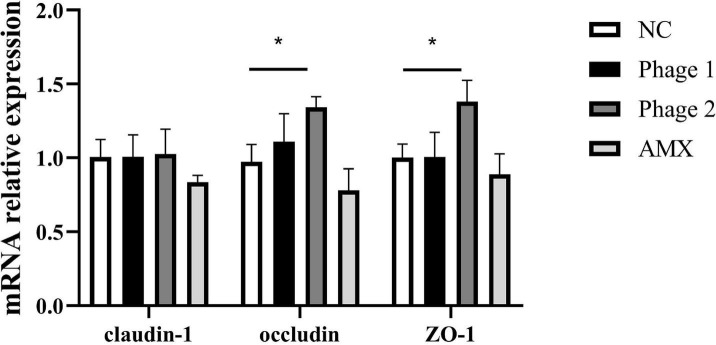
Effect of phages on the relative mRNA expression of the jejunal tight junction. Fold induction of relative mRNA expressions of claudin-1, occludin, and ZO-1 (*n* = 5). NC, negative control; Phage 1, 10^9^PFU GRNsp6 (day 3-5); Phage 2, 10^9^PFU GRNsp6 (day 8-10); AMX, 1 mg/ml amoxicillin (day 8-10). *p < 0.05.

Tight junctions are a major determinant of intercellular permeability; therefore, the expressions of claudin-1, occludin, and ZO-1 in the jejunum were detected. We observed a significant increase (*p* < 0.05) in the mRNA expression of occludin and ZO-1 in the Phage 2 group, but did not detect significant differences in claudin-1, occludin, and ZO-1 mRNA expression in the Phage 1 group (Figure 3).

### Serum Endotoxin Concentration and Relative mRNA Expression of Jejunal Inflammatory Cytokines

**FIGURE 4:**
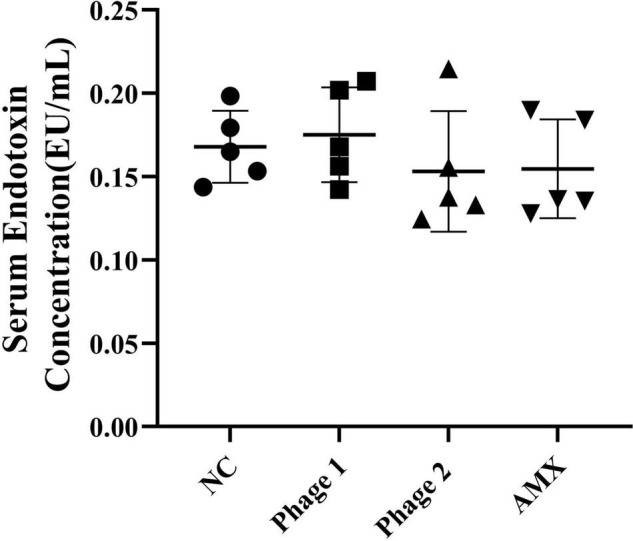
Effect of phage on serum endotoxin in chicks (*n* = 5). The serum endotoxin level is an important biomarker of intestinal permeability and was detected using an ELISA kit. NC, negative control; Phage 1, 10^9^PFU GRNsp6 (day 3-5); Phage 2, 10^9^PFU GRNsp6 (day 8-10); AMX, 1 mg/ml amoxicillin (day 8-10).

**FIGURE 5:**
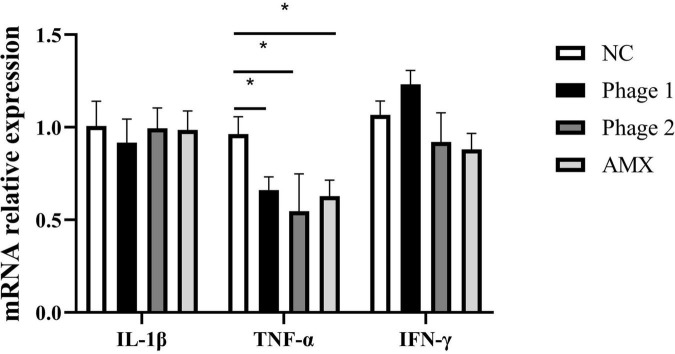
Effect of phages on the relative mRNA expression of jejunal cytokines. Fold induction of relative mRNA expressions of IL-1β, TNF-α, and IFN-γ (*n* = 5). NC, negative control; Phage 1, 10^9^PFU GRNsp6 (day 3-5); Phage 2, 10^9^PFU GRNsp6 (day 8-10); AMX, 1 mg/ml amoxicillin (day 8-10).*p < 0.05.

Intestinal permeability, related to intestinal barrier function, depends on the integrity of the intestinal mucosal barrier. Endotoxin, a critical bacterial antigen in the cell wall of gram-negative bacteria, is released into the circulation with impaired gut permeability. Thus, we measured serum endotoxin concentrations in chicks. Figure 4 shows that the ingestion of phages did not cause elevated (*p* > 0.05) serum endotoxin concentrations. This suggests that phages did not damage the intestines of chicks by disrupting intestinal permeability during intestinal development. In addition, phages, which are protein-encapsulated viruses, may cause inflammatory responses and damage the intestinal tissues. Subsequently, the relative expression levels of three inflammatory cytokines (IL-1β, TNF-α, and IFN-γ) in the jejunum were determined. We found that the administration of bacteriophages or amoxicillin significantly decreased (*p* < 0.05) TNF-α mRNA expression compared to that in the NC group (Figure 5).

### Bacteriophages Affect Intestinal Microbial Communities

**FIGURE 6:**
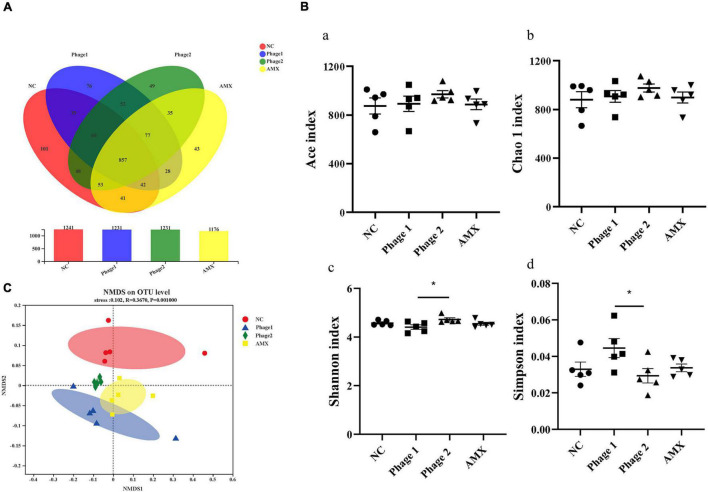
Effect of phages on the cecal microbiota of chicks. **(A)** Venn diagram generated to compare operational taxonomic units (OTUs) between different groups. **(B)** Diversity and richness indexes of cecal microbiota in each group. Figure a-d represents ACE, Chao1, Shannon and Simpson indices, respectively. *p < 0.05. **(C)** Beta-diversity analysis of cecal microbiota among groups. NMDS analysis based on Bray–Curtis distances. Points of different colors or shapes represent samples of different groups. The closer the two sample points are, the more similar the species composition of the two samples. NC, negative control; Phage 1, 10^9^PFU GRNsp6 (day 3-5); Phage 2, 10^9^PFU GRNsp6 (day 8-10); AMX, 1 mg/ml amoxicillin (day 8-10).

The cecum contains the largest number and variety of bacteria in the chicken gastrointestinal tract and is the main site of microbial fermentation. We further investigated the effects and regulatory roles of phages targeting *Salmonella* in cecal bacterial communities. The cecum contents of chicks from the NC, Phage 1, Phage 2, and AMX groups contained 1,241, 1,231, 1,231, and 1,176 OTUs, respectively. A total of 857 common bacteria and 263 individual bacteria were isolated from the four groups (Figure 6A). Notable, different processing methods can lead to unique OTU appearances. In addition, using Shannon, Simpson, Chao1, and ACE indices, we performed alpha diversity analysis between groups (Schloss et al., 2009). No significant differences in ACE and Chao1 indices were observed (Figure 6B). However, the diversity estimator (Shannon) of the Phage 1 group was significantly lower than that of the Phage 2 group, and the Simpson indices of the Phage 1 group were significantly higher than those of the Phage 2 group (Figure 6B). Subsequently, the similarity of the microbial community structure was evaluated by NMDS analysis at the OTU level, as shown in Figure 6C. We found that the NC, Phage 1, Phage 2, and AMX groups clustered separately (*p* = 0.001). As shown in Figure 6C, there was a greater difference in microbial community composition between the NC and Phage 1 groups compared to the other groups. These data demonstrate that oral administration of high doses of single phages did not significantly affect alpha diversity, but the bacterial community composition was significantly influenced. Community diversity and composition correlate with the timing of phage administration during the establishment and development of the intestinal microflora of chicks.

### Microbial Community Structure Analysis

**FIGURE 7:**
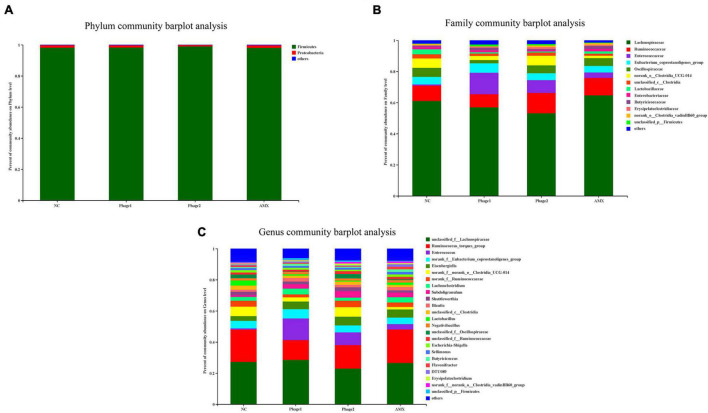
Effect of phages on the microbiota composition in the cecal digesta at different taxonomic levels **(A)** at phylum levels, **(B)** at family levels, and **(C)** at genus levels). Less than 1% abundance at the phylum, family, or genus level was merged with others. NC, negative control; Phage 1, 10^9^PFU GRNsp6 (day 3-5); Phage 2, 10^9^PFU GRNsp6 (day 8-10); AMX, 1 mg/ml amoxicillin (day 8-10).

At the phylum level, the most dominant bacteria were *Firmicutes*, accounting for more than 95% of the entire flora (Figure 7A). *Lachnospiraceae* and *Ruminococcaceae* were the predominant members of *Firmicutes* (Figure 7B). At the genus level, administration of bacteriophages altered the community composition. The relative abundances of *Enterococcus* (13.8%, 8.2%) of the Phage 1 and Phage 2 groups were found to be higher than those of the NC group (0.8%), and that of the *Ruminococcus*_torques_group (13.7%, 15.1%) was lower than that of the control (20.9%) (Figure 7C).

**FIGURE 8:**
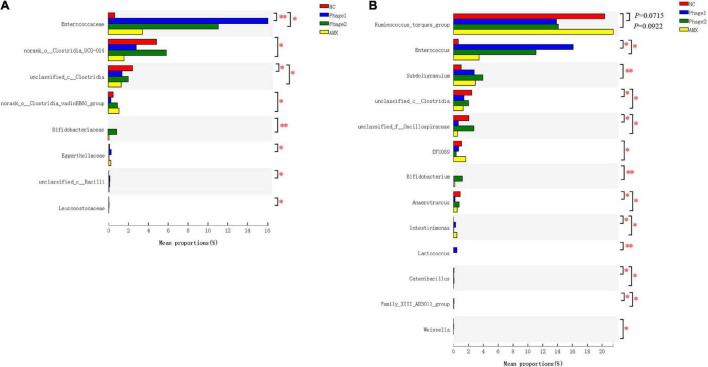
Effect of phages on the relative bacterial abundance in chicks. Analysis of the differential flora of cecal microbiota at **(A)** family level and **(B)** genus level. The Kruskal–Wallis H test was used for multi-group comparison of cecal microorganisms, and Scheffe *post hoc* tests were used to test the difference between any two groups. NC, negative control; Phage 1, 10^9^PFU GRNsp6 (day 3-5); Phage 2, 10^9^PFU GRNsp6 (day 8-10); AMX, 1 mg/ml amoxicillin (day 8-10). *p < 0.05; ^**^p < 0.01; and *p* < 0.1 trend.

Moreover, we explored in more depth the differences in relative abundance of bacteria with multi-group comparison and LEfSe analysis. Oral administration of phages significantly increased the relative abundance of *Enterococcaceae* (Figure 8A) and tended to decrease (0.05 < *p* < 0.1) the abundance of *Ruminococcus*_torques_group. In addition, Phage 1 samples showed an increase (*p* < 0.05) in *Lactococcus* (*p* < 0.01), Family_XIII_AD3011_group (*P* < 0.05), and *Intestinimonas* (*p* < 0.05), and simultaneously a decrease (*p* < 0.05) in unclassified_f__*Oscillospiraceae*, unclassified_c__*Clostridia, Catenibacillus*, and *Anaerotruncus*. A decrease in the abundance of these four bacteria was observed in the AMX group. In addition, Phage 2 samples revealed that the number of *Bifidobacterium* and *Subdoligranulum* significantly increased (*p* < 0.01). Notably, *Intestinimonas* and DTU089 were more abundant (*p* < 0.05) in the AMX group, whereas the populations of *Weissella* (*p* < 0.05) were less abundant (Figure 8B).

**FIGURE 9:**
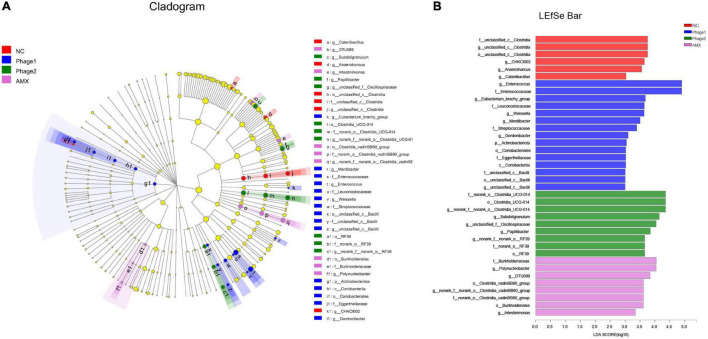
LEfSe analysis of cecal microbiota. Cladogram **(A)** and LDA scores **(B)** show taxa that best characterize each biological class. Different-colored regions represent different constituents. Circles indicate phylogenetic level from domain to genus. The diameter of each circle is proportional to the abundance of the group. Species with significant difference that have an LDA score greater than the estimated value (3.0). The length of the histogram represents the LDA score. NC, negative control; Phage 1, 10^9^PFU GRNsp6 (day 3-5); Phage 2, 10^9^PFU GRNsp6 (day 8-10); AMX, 1 mg/ml amoxicillin (day 8-10).

LEfSe analysis was applied to identify specialized communities (*p* < 0.05, LDA > 3.0) in the four groups. As shown in Figure 9, these results confirmed the significant enrichment of *Clostridia*_UCG-014 (from order to genus) and unclassified_c__*Clostridia* (from order to genus) in the NC group. Unclassified_p__*Firmicutes* (from class to genus), *Lactobacillales*, *Enterococcaceae* (from family to genus), *Leuconostocaceae* (from family to genus), and *Christensenellaceae* (from family to genus) were enriched in the Phage 1 group. In the Phage 2 group, RF39 (from order to genus), *Papillibacter*, and *Catenibacillus* were significantly enriched. The microbiota from the AMX group was differentially enriched with *Actinobacteria*, *Coriobacteriia* (from class to genus), *Lachnospirales* (from order to genus), and *Clostridia*_vadinBB60_group (from order to genus).

### Functional Prediction of Cecal Microbiota

**FIGURE 10:**
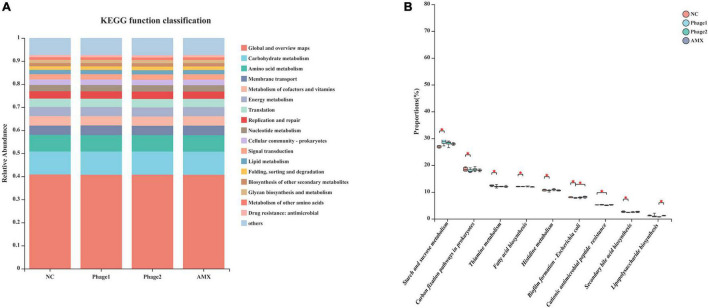
Functional prediction of cecal microbiota **(A)** KEGG Pathway level 2 function classification; **(B)** Differential analysis of the KEGG pathway level 3 of cecal microbiota. The Kruskal–Wallis H test was used for differential analysis. NC, negative control; Phage 1, 10^9^PFU GRNsp6 (day 3-5); Phage 2, 10^9^PFU GRNsp6 (day 8-10); AMX, 1 mg/ml amoxicillin (day 8-10). *p < 0.05.

PICRUSt2 was used to calculate the gene content from the 16S rRNA gene data and to analyze the functional potential of the cecal microbiota. According to KEGG prediction, certain biological processes, such as carbohydrate metabolism, membrane transport, amino acid metabolism, energy metabolism, and metabolism of cofactors and vitamins were enriched in the four groups (Figure 10A). Figure 10B shows that the cecal microbiota of the Phage 1 group displayed a lower (*p* < 0.05) abundance of functions involving metabolic pathways (histidine metabolism, beta-alanine metabolism, fatty acid biosynthesis, thiamine metabolism, carbon fixation pathways in prokaryotes, and secondary bile acid biosynthesis) and a higher (*p* < 0.05) abundance of functions, such as starch and sucrose metabolism and biofilm formation – *E. coli*, than those in the NC group. In addition, we observed that the cecal microbiota of the Phage 2 group displayed a lower (*p* < 0.05) abundance of cationic AMP resistance than the NC group.

## Discussion

Excessive use of antibiotics not only accelerates the spread of bacterial resistance but also leads to an imbalance in intestinal microbes and causes antibiotic-associated diarrhea (Leffler and Lamont, 2015). Additionally, phages have even been added as a feed additive to improve the growth of broilers in response to increasing bacterial resistance (Upadhaya et al., 2021). Considering the current limitations of the use of antibiotics in poultry farming, we screened a *Salmonella*-targeting phage strain that does not encode lysogenic and drug-resistant genes and had a broad spectrum of lysis in a previous study (not yet published). This study aimed to investigate the effects of high-dose oral bacteriophages on the intestinal barrier, focusing on how *Salmonella* phage targeting affects the cecal microbiota of chicks.

During the experiment, oral administration of high doses of phages to chicks at 3 and 8 days of age had no significant effect on body weight compared with the NC group. First, this may be related to the short trial period. Secondly, this may also be responsible for the rapid uptake of phages by the reticuloendothelial system in the liver and kidney when host bacteria are absent or present in small numbers (Geier et al., 1973; Merril et al., 2003). Antibiotics cannot be completely absorbed by animals and have a high residual concentration in animal feces (Muhammad et al., 2019), which may cause the generation and spread of resistant bacteria and resistance genes (Levy et al., 1976), resulting in considerable negative effects on ecosystem security and human health (Manzetti and Ghisi, 2014). Therefore, it may be safer and more environmentally friendly to use phages at the farm-side rather than antibiotics.

The intestinal barrier prevents harmful agents such as pathogens and endotoxins from entering the bloodstream, and is an important barrier for maintaining the stability of the internal environment of the body. In the early stages of growth, the intestinal tract of chickens is not fully developed and easily affected by exogenous substances. We administered bacteriophages, a type of bacterial virus, to chicks in an oral form that directly contacted the gut of the animal. H&E staining and qPCR results showed that the phage did not destroy the intestinal tight junction, did not cause intestinal inflammation, or result in morphological damage to the small intestine. Intestinal morphology is considered an important parameter reflecting intestinal health and recovery. Administration of bacteriophages increased the jejunal height of the villus, the ratio of V/C, and the mRNA levels of occludin and ZO-1 in the Phage 2 group, which may suggest that oral phages enhance intestinal barrier function under certain conditions. The difference between the Phage 1 and 2 groups may be related to the different timing of the gavage phages. In addition, we observed that oral phages could downregulate the expression of TNF-α mRNA in the jejunum of chicks. Previous studies have shown that phages inhibit inflammation in ways other than killing disease-causing bacteria (Miernikiewicz et al., 2013; Van Belleghem et al., 2017; Zhang et al., 2018). Our experiment also showed the ability of phages to induce anti-inflammatory properties unrelated to their antibacterial activity. Phages are prokaryotic viruses that do not interact directly with eukaryotic cells. We speculate that these changes may be related to bacteriophage-induced changes in intestinal flora.

The serum endotoxin content has been considered an indicator of the extent of intestinal damage (Mani et al., 2019). A higher serum endotoxin content was not observed in the challenged chicks, suggesting that the oral administration of phages did not impair intestinal barrier function, which is inconsistent with a previous study (Tetz et al., 2017). On the one hand, we used a single highly purified phage strain, excluding the effect of bacterial endotoxins and proteins on intestinal permeability, and on the other hand, the commercial cocktail used by Tetz et al. (2017) contained a variety of phages targeting *Enterobacteriaceae*, *Staphylococcaceae*, *Streptococcaceae*, and *Pseudomonadaceae*, which may extensively lyse bacteria in the intestine, releasing large amounts of lysis products, causing disorders of intestinal flora, thus disrupting intestinal permeability. We reasoned that the phage particles themselves did not affect the intestinal permeability and compromised the intestinal barrier. However, phages can indirectly affect the composition of the intestinal flora by directly lysing bacteria in the intestine, ultimately affecting intestinal health. The ingestion of large numbers of different species of phages can cause disturbances in the intestinal flora, and they may become potential pathogenic factors in animals.

In recent years, phages have been considered potential microbial modifiers for promoting intestinal health. The host specificity of phages offers the possibility of selective alteration of gut microbiota without causing dysbiosis of the microbial community. Thus, the focus of this study was to analyze the cecal microbiota of chicks. We confirmed that bacteriophage challenge did not affect microbial alpha diversity due to the lack of differences in community richness and diversity. However, clustering of groups was apparent in an NMDS plot, and the distance between the Phage 1 and NC groups was the largest. The greatest differences in community structure and composition were found between the Phage 1 and NC groups, as evidenced by the results of LEfSe analysis and Kruskal–Wallis H tests; the highest number of differential bacteria was found in the Phage 1group. The magnitude of the changes in the composition of the cecal microbiota correlated with the time of phage use, which is also supported by the significant difference in Simpson and Shannon indices between Phage 1 and 2 groups. This may be attributed to the weak resistance and stability of the early intestinal microbiota of poultry (Rubio, 2019),which is susceptible to external disturbances. Videnska et al. (2014) divided the development of the cecal microbiota of chickens into four stages. The first stage lasted for the first week of chicken life and was characterized by *Enterobacteriaceae*. The second stage lasted from week 2 to 4 and was characterized by the absolute dominance of the families *Lachnospiraceae* and *Ruminococcaceae* (Videnska et al., 2014; Ballou et al., 2016; Richards P. et al., 2019), which is consistent with the results of our study. The first two phases are the periods of the most dramatic changes in microbial composition and the window of opportunity for the regulation of intestinal microbes (Gong et al., 2019; Jurburg et al., 2019; Kogut, 2019; Wilson et al., 2019). Therefore, gut microbes are vulnerable to external factors. Bacteriophages targeting *Salmonella* have the potential to lyse *E. coli* across genera. Thus, in the first stage, dominated by *Enterobacteriaceae*, *Salmonella* phages may have a more pronounced effect on cecal microbes, which may account for the significant changes in the cecal microbial community composition of chicks in the Phage 1 group.

The transition from the initial microbiota to the mature microbiota of newborn chicks is also a critical period for the development of the intestinal tract and immune system, which is highly susceptible to bacterial diseases, such as *Salmonella*. At the family level, samples of cecal contents from all chicks fed phages showed a decrease in *Lachnospiraceae* and an increase in *Enterococcaceae* (Figure 7B). Thus, the increased *Enterococcaceae* abundance may be caused by phages that target *Salmonella*. Previous studies have shown that *Enterococcus* plays a beneficial role in the jejunal morphology of chickens (Samli et al., 2007, 2010). It has been found that certain strains of *Enterococcus* can inhibit pathogenic bacteria proliferation, including *Salmonella* and *E. coli* (Levkut et al., 2012; Cao et al., 2013; Huang et al., 2018; Zhang et al., 2021). This implies that phages may defend against pathogenic bacteria by both direct lysis of target bacteria (Fiorentin et al., 2005) and regulation of the intestinal flora. Additionally, we believe that lactic acid produced by *Enterococcus* is responsible for the significant decrease in TNF-α mRNA expression in the jejunum. However, *Enterococcus* spp. are opportunistic pathogens, and some strains even cause death in chicks. Although we did not observe adverse effects of the elevated abundance of *Enterococcus* caused by phages in chicks, new experiments must be designed to examine its safety.

Notably, the phage targeting *Salmonella* used in this experiment had no direct effect on the genera whose abundance was significantly altered. This is in agreement with previous studies, which have shown that the microbiota is characterized by a dynamic equilibrium, and its variation can lead to complex and unpredictable consequences (Blaser and Kirschner, 2007). Furthermore, the idea that phages induce cascading effects on microbiota species that are not directly targeted was raised (Hsu et al., 2019). The use of phages at different stages of the development of the gut microbiota leads to different results. For example, LEfSe analysis showed that *Christensenellaceae* were enriched in the Phage 1 group. According to the Kruskal–Wallis H test, the Phage 2 group also showed a significant increase in *Subdoligranulum* and *Bifidobacterium*. *Subdoligranulum*, a butyrate-producing bacterium (Holmstrom et al., 2004), is a promising probiotic candidate that positively correlates with gut integrity and multiple beneficial health effects on host energy metabolism (Leclercq et al., 2014; Van Hul et al., 2020). *Bifidobacteria* can regulate the composition of the intestinal flora and improve the integrity of the intestinal mucosa. Ewaschuk (Ewaschuk et al., 2008) reported that it also promotes tight junction protein expression in intestinal epithelial cells. This may be the reason why we observed a significant difference in the jejunal height of the villus and the ratio of V/C and mRNA expression of jejunal tight junctions in the Phage 2 group. Finally, compared with the NC group, we did not observe certain low-abundance bacteria, such as *Weissella*, in the AMX group. In the analysis of intergroup differences, the number of differential flora in the AMX group was second only to that in the Phage 1 group. This also confirmed our hypothesis that phages significantly reduce the impact of cecal microbiota on chicks compared to broad-spectrum antibiotics.

Finally, we used PICRUSt2 to infer the functional relevance of changes in the gut microbiome composition. Kruskal–Wallis H test results showed that most of the differences between groups were concentrated in the Phage 1 and NC groups, which means that oral administration of high doses of phage to chicks before one week of age may affect the metabolic level of the organism. This suggests that caution should be exercised regarding the use of phages. Notably, compared to amoxicillin, orally administered phages can inhibit some regulatory pathways, namely biofilm formation – *E. coli* and lipopolysaccharide biosynthesis. Further studies are needed to determine whether phages are superior in inhibiting biofilm formation and lipopolysaccharide synthesis.

Although we tried to eliminate some influencing factors (environment, feeding management, and nutritional intake), the gut microbes can be disturbed by factors beyond our control. Unfortunately, due to the short experimental period, no effect of microbiome changes on the subsequent development of chicks was observed, and we did not study the effects of different doses of phages on the cecal microbiome in chicks; nevertheless our experiments demonstrated that phages can interfere with the microbiota of chicks, which may provide new ideas for phage resistance to bacterial infections in the intestine. We will continue to verify the role of changes in the intestinal flora of chickens caused by *Salmonella* phages for protection against *Salmonella* in subsequent experiments.

## Conclusion

Oral phage-induced changes in intestinal barrier function may be related to changes in the intestinal flora induced by phages. The magnitude of the changes in the composition of the intestinal flora correlates with the duration of phage use. *Salmonella* phages can easily cause substantial changes in the intestinal flora, especially during the first stage of cecal microbiome development. The results of this study suggest that we should be cautious about the period of phage use.

## Data Availability Statement

Raw sequence data are deposited in the NCBI database within the Bioproject PRJNA838399.

## Ethics Statement

The animal study was reviewed and approved by Animal Experimental Ethical Inspection of the Laboratory Animal Center, Huazhong Agricultural University.

## Author Contributions

XW and HZ contributed to the concept and method of the article. HZ obtained the article data and drafted the manuscript. YL provided assistance for data analysis of the article. PL, JH, and RT contributed the sample collection and reagent preparation. XJ, JW, and XW reviewed the manuscript. All authors read and approved the published version of the manuscript.

## Conflict of Interest

The authors declare that the research was conducted in the absence of any commercial or financial relationships that could be construed as a potential conflict of interest.

## Publisher’s Note

All claims expressed in this article are solely those of the authors and do not necessarily represent those of their affiliated organizations, or those of the publisher, the editors and the reviewers. Any product that may be evaluated in this article, or claim that may be made by its manufacturer, is not guaranteed or endorsed by the publisher.
